# “I am not delusional!” Sensory dysaesthesia secondary to degenerative cervical myelopathy

**DOI:** 10.1136/bcr-2018-229033

**Published:** 2019-04-11

**Authors:** Oliver Daniel Mowforth, Benjamin Marshall Davies, Mark Reinhard Kotter

**Affiliations:** 1 Division of Neurosurgery, Department of Clinical Neurosciences, University of Cambridge, Cambridge, UK; 2 Anne McLaren Laboratory for Regenerative Medicine, Wellcome-MRC Cambridge Stem Cell Institute, University of Cambridge, Cambridge, UK

**Keywords:** spinal cord, pain (neurology), neurology, neurological injury

## Abstract

Degenerative cervical myelopathy (DCM) is the most common cause of adult spinal cord dysfunction, most classically presenting with a broad-based gait and clumsy hands. Limb sensory loss and paraesthesia are considered common symptoms of DCM. However, we report an unusual case of a patient presenting with prominent and atypical sensory symptoms. The patient repeatedly presented to accident and emergency complaining of her body resembling a wet gel-like substance that she attributed to the use of olive oil moisturising cream. The patient was found to have myelopathic signs on examination and MRI consistent with severe cervical myelopathy. She subsequently underwent successful decompressive anterior cervical discectomy, as recommended by international guidelines. This case serves to remind health professionals of uncommon presentations of common disease and the importance of maintaining a wide initial differential diagnosis.

## Background

The detection of degenerative cervical myelopathy (DCM), particularly early detection, is challenging^1^ and currently contributes to long average diagnostic delays of 2.2 years^2^ and poorer response to treatment.^3^

Patients with textbook descriptions of DCM present with a range of disabling symptoms^1^ : limb pain, weakness, stiffness and numbness are prevalent; neck stiffness and neck pain, loss of manual dexterity, imbalance, gait problems and falls are commonly reported. Patients also experience bladder and bowel dysfunction. However, recent studies have described the importance of ‘atypical’ symptoms, such as respiratory dysfunction, chest tightness and headaches. For example, one study found that the OR of chest tightness in myelopathy patients compared with controls was 22.9.^4^

This case provides an example of an unusual presentation of this common disease. It highlights the frustrations faced by patients in reaching a diagnosis, the numerous consultations required and the progression of symptoms in the interim. It serves to reiterate the importance of taking a full history and performing a comprehensive neurological examination in any patient presenting with possible neurological symptoms before diagnosing a disorder as non-organic.

## Case presentation

A 62-year-old woman was referred to our neurosurgical outpatient service with abnormal sensation in her trunk, arms and legs. The patient had a past medical history of a gastric ulcer, a right ankle plating after fracture 20 years ago and a headlice infection 6 months before first presentation. She was a smoker, social drinker of alcohol and was not taking any regular medication. She lived alone and had been struggling to manage independently.

Since her symptoms commenced 3 years previously, the patient had presented to emergency department (ED) on 11 occasions. Her primary complaint was of dysaesthetic sensory symptoms including a feeling of water retention and a gel infiltrating the skin of her face, trunk, arms and legs, feeling there was something stuck on her skin and feeling her hair was stuck down. In the weeks before the onset of her symptoms, the patient started using an olive oil moisturising cream, to which she attributed her symptoms.

While dermatological examination was conducted, comprehensive neurological examinations were not documented during the first presentations to the ED, which focused on the patient’s facial dysaesthesia.

The patient was frustrated that her symptoms were repeatedly dismissed as delusional by the ED staff. She refused assessment by liaison psychiatry and mental health review by her general practitioner. The patient was felt to have capacity throughout all consultations. The patient was not taking any psychiatric medications.

Over time her symptoms progressed. She started to complain of back pain, multiple falls and episodes of her right leg giving way which had progressed to severely compromised walking and coordination. The patient also complained of episodes of urinary and faecal incontinence. This led to a lumbar MRI which ruled out cauda equina syndrome. In her final presentation before referral, she also complained of stiff legs, difficulty walking, difficulty passing urine, reduced manual dexterity and neck pain. A neurology assessment was finally sought and an MRI for suspected myelopathy was organised.

On presentation to our neurosurgical clinic, the patient’s complaint remained sensory dysaesthesia from her neck down, particularly affecting her hands and groins. She complained of her body feeling like a ‘wet gel-like substance’. In addition to the above symptoms, the patient also complained of a 3-year medical history of numbness and tingling in her upper limbs.

On examination in the neurosurgical clinic, the patient had hyperaesthesia from the neck down, most prominent in the hands and groins. The patient had reduced grip strength of 3/5, finger extension and abduction of 3/5, wrist flexion and extension of 3/5, elbow flexion of 4+/5, elbow extension of 4/5 and shoulder abduction of 4+/5 on the Medical Research Council power scale. Hip flexion was 3/5, with all other muscle groups in the lower limb at 4+/5. The patient had very brisk reflexes with pathological spreading in the upper and lower limbs. There was self-limiting clonus in the ankle bilaterally. Hoffmann’s and Babinski reflexes were positive bilaterally. There was a severe loss of coordination and balance. Gait was impaired; the patient required a frame to mobilise. The severity of the patient’s cervical myelopathy was scored as 1 (upper limb motor dysfunction) +3 (lower limb motor dysfunction) +1 (upper limb sensory dysfunction) +1 (sphincter dysfunction) =6 (severe myelopathy) on the modified Japanese Orthopaedic Association scale (mJOA).

## Investigations

**Figure 1:**
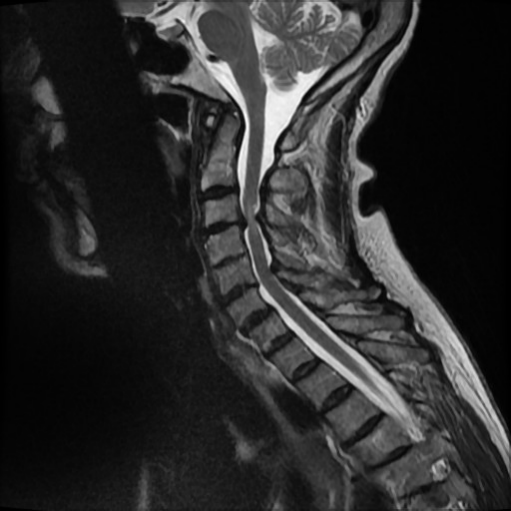
Sagittal MRI of cervical spine.

The patient underwent MRI imaging and was found to have multilevel degenerative changes of the cervical spine. At C3/4 there was a broad-based disc osteophyte protrusion causing compression of the spinal cord with T2 hyperintense signal changes within the cord. At C5/6, there was a broad-based disc osteophyte protrusion with mild narrowing of the C6 neural foramina (figure 1).

## Differential diagnosis

In addition to DCM, the case highlights a variety of differentials:

1. Delusional disorder.
2. Trichotillomania.
3. Cauda equina syndrome.

A possible differential was that the patient’s sensory symptoms were independent of her myelopathy and the result of a sensory delusional disorder. Alternatively, comorbid mild psychiatric illness could have caused the patient to interpret her myelopathic symptoms in an unusual way.

However, there was no history of formally-diagnosed psychiatric illness or evidence of previous complaints of psychiatric aetiology in this 62-year-old patient. In fact, the patient had a strong conviction that the onset of symptoms around the time of her change in moistening cream was not coincidental and was frustrated that her ideas were being dismissed as psychological by some health professionals. Her anger at these suggestions were further interpreted as being evidence of an underlying psychological condition.

## Treatment

The patient was found to be suffering from severe cervical myelopathy which required expedited surgical decompression. She underwent anterior cervical discectomy at C3/4.

## Outcome and follow-up

The patient was recovering well on review 3 months post-surgery. Cervical spine radiographs demonstrated appropriate position of her spinal implants. Her wound had healed well. She reported reduced sensory problems and reduced paraesthesia in her fingers. On examination, compared with pre-surgery power, grip strength had improved to 4+/5, wrist flexion and extension had improved to 4/5 and elbow flexion, extension and shoulder abduction had improved to 5/5. Finger extension and abduction remained 3/5. Power had improved to 5/5 in all muscle groups in the patient’s lower limbs. The remainder of the examination was unchanged.

In addition, at 9 months the patient had an improved mJOA of 10 (3+3+2+2) and she reported improved manual dexterity, improved walking, improved bladder function and resolution of incontinence. The dysaesthesia affecting her body was still present, although reduced.

Full recovery is unexpected following surgery for DCM, with current evidence suggesting that surgery is highly effective in halting disease progression but has limited benefit in reversing neurological damage already present.^3^ Thus, the incomplete resolution of her dysaesthetic sensory symptoms is not unexpected.

## Discussion

DCM is defined as symptomatic spinal cord compression secondary to degenerative changes in the cervical spine.^5^ It is common with a prevalence of up to 5% in the over 40s,^1^ which is expected to rise as populations age.^4 5^ However, most patients never get a diagnosis,^6 7^ and those that do face long diagnostic delays, often with initial misdiagnosis.^2^ This is significant given that surgery is the only currently-effective treatment but cannot reverse existing damage.^3 8 9^

Misdiagnosis and diagnostic delay are key problems in DCM. As in this case, patients often consult many times and wait years for accurate diagnosis.^2^ The reasons for this are unclear, but likely include a poor awareness of DCM among non-specialist health professionals,^1^ subtle non-specific early symptoms^10^ and poor current understanding of the most common symptoms and natural history.^11^ Substantial diagnostic delays in the context of the progressive nature of DCM is a likely key factor behind DCM having patient quality of life scores among the worst of any chronic disease.^12^

Sensory dysaesthesia is defined as abnormal sensation. The involvement of all four limbs in our patient is consistent with a myelopathic aetiology, however we are unaware of previous reports of such widespread sensory dysaesthesia in DCM. Nonetheless, unusual sensory presentations of other common neurological diseases, such as Guillain–Barré syndrome,^13^ have been reported.

While facial sensory dysaesthesia would be unexpected secondary to spinal pathology, recent studies report structural and functional changes rostral to the compressive lesion in DCM.^14 15^ For example, one recent study reported a correlation between depression and activity in the anterior cingulate cortex (ACC) in DCM patients^16^; spinal decompressive surgery alleviated depression and diminished activity in the ACC. While the precise mechanism remains to be elucidated, cortical reorganisation secondary to reduced efficiency of signal transduction through the compressed segments of spine is plausible. Cortical reorganisation is thus a possible explanation for the facial component of the patient’s dysaesthesia.

Unfortunately, eccentric interpretation of aberrant sensation secondary to myelopathy was misinterpreted by health professionals as delusional. At no point in the 11 consultations by doctors ranging from foundation year to consultant level was myelopathy documented as a differential diagnosis. Given that the patient’s myelopathy was severe at time of eventual diagnosis and in the absence of other pathology, it is likely myelopathy was causing the dysaesthetic symptom onset 3 years previously.

In conclusion, this case illustrates a novel and unusual example of a common scenario for DCM patients: multiple consultations, diagnostic delay, symptom progression, greater disability and poorer response to surgery after eventual diagnosis. To address these issues, a high index of suspicion, an awareness of the progressive nature of DCM and a comprehensive neurological assessment in any patient with neurological symptoms in the hands and legs are required.

**Learning points**

- Degenerative cervical myelopathy (DCM) is a common neurological syndrome.
- DCM is progressive, typically affecting patients over 40 years old, although younger presentations are possible.
- DCM can present with a range of typical symptoms and should be considered in patients presenting with any neurological symptoms in their limbs or neck.
- Awareness, a high index of suspicion, comprehensive neurological assessment and a low threshold for obtaining a cervical MRI are required for early diagnosis.
